# Weight progression and adherence to weight gain target in women with vs. without gestational diabetes: a retrospective cohort study

**DOI:** 10.1186/s12884-023-05832-x

**Published:** 2023-07-13

**Authors:** Hong Miao, Feng Liang, Zheng Zheng, Huimin Chen, Xiaojun Li, Yi Guo, Kuanrong Li, Xihong Liu, Huimin Xia

**Affiliations:** 1 Clinical Data Center, Institute of Pediatrics, Guangzhou Women and Children’s Medical Center, Guangzhou Medical University, 9 Jinsui Road, Guangzhou, 510623 Guangdong China; 2 Department of Obstetrics, Guangzhou Women and Children’s Medical Center, Guangzhou Medical University, Guangzhou, 510623 Guangdong China; 3 Department of Clinical Nutrition, Guangzhou Women and Children’s Medical Center, Guangzhou Medical University, Guangzhou, 510623 Guangdong China; 4 Guangdong Provincial Key Laboratory of Research in Structural Birth Defect Disease, Guangzhou Women and Children’s Medical Center, Guangzhou Medical University, Guangzhou, 510623 Guangdong China

**Keywords:** Gestational diabetes mellitus, Weight progression, Gestational weight gain targets, Overweight and obesity

## Abstract

**Background:**

Weight management has been an important component of the service in obstetric care offered to pregnant women. Current gestational weight gain recommendations were primarily for the general obstetric population, raising concern about the applicability to women with gestational diabetes mellitus (GDM). We aimed to assess the difference in weight progression and adherence to the recommended gestational weight gain targets between women with gestational diabetes mellitus (GDM) and women with normal glucose tolerance (NGT).

**Methods:**

This was a hospital-based retrospective study of 56,616 pregnant women (9,430 GDM women and 47,186 NGT women) from Guangzhou between 2017 and 2021. The average change in weight progression was estimated based on serial weight measurements throughout pregnancy, using a mixed effects model with a random intercept to account for repeated measures of the same individual.

**Results:**

Women with GDM gained less weight (12.07 [SD 5.20] kg) than women with NGT (14.04 [SD 5.04] kg) throughout pregnancy. Before OGTT, a small difference was observed in the average change in weight progression between the two groups (GDM, 0.44 kg/week vs. NGT, 0.45 kg/week, p < 0.001), however, this gap widened significantly after the test (0.34 vs. 0.50 kg/week, p < 0.001). GDM individuals were identified with an approximately 4-fold increased proportion of insufficient weight gain (41.1% vs. 10.4%) and a 2-fold decreased proportion of excessive weight gain (22.6% vs. 54.2%) compared to NGT individuals. These results were consistently observed across different BMI categories, including underweight (insufficient: 52.7% vs. 19.9%; excessive: 15.6% vs. 35.3%), normal weight (insufficient 38.2% vs. 7.4%; excessive: 22.2% vs. 57.3%), and overweight/obese (insufficient: 43.1% vs. 9.8%; excessive: 30.1% vs. 68.8%).

**Conclusion:**

Weight progression varied significantly between GDM and NGT individuals, resulting in a substantial difference in identifying insufficient and excessive weight gain between the two groups under current gestational weight gain guidelines.

**Supplementary Information:**

The online version contains supplementary material available at 10.1186/s12884-023-05832-x.

## Introduction

Gestational weight gain has been recognized as a crucial modifier factor for maternal and fetal outcomes [1–4]. Inappropriate gestational weight gain (GWG), especially excessive GWG, has been linked to a variety of short- and long-term adverse health consequences for mothers and their offspring, including the increased risks of gestational diabetes mellitus (GDM) [5, 6], fetal macrosomia [7, 8], obesity, and adverse cardiometabolic profiles in the offspring [9–12].

In 2009, the Institute of Medicine (IOM) published national guidelines for GWG [13]. These guidelines established specific recommendations stratified by pre-pregnancy BMI class and were quickly adopted widely in many countries [13]. In China, weight management has been an important component of the service in obstetric care offered to pregnant women. Recently, given the ethnic variation in body mass and stature, the Chinese nutrition society released lower GWG and GWR targets for the Asian population in October 2021 [14]. However, both guidelines were primarily for the general obstetric population and do not differentiate between women with normal glucose tolerance (NGT) and those with gestational diabetes mellitus (GDM). GDM is an increasingly common chronic disease in pregnancy that affects about 15% of pregnant women [15, 16]. Women affected by GDM usually receive more intensive and comprehensive health interventions than those with NGT as a part of the glycemic control program [17–19]. As a result of these interventions, it can be hypothesized that gestational weight may progress in distinctly different ways between women with GDM and those with NGT. In previous studies, it was noted that women with GDM gained fewer amounts of weight [20, 21] than the general obstetric population through pregnancy [22–24]. However, it is unknown how gestational weight changes during pregnancy and to what extent the weight progression differs between the two groups.

The objective of this study was to assess the difference in weight progression between women with GDM and NGT in the total cohort and across different BMI categories. A further objective was to evaluate adherence to the current gestational weight guidelines for these two groups.

## Materials and methods

### Study design and population

This hospital-based retrospective cohort study was conducted at the Guangzhou Women and Children’s Medical Center (GWCMC), Guangzhou, south China [25], which serves in general about 100,000 pregnant women every year with comprehensive obstetrical care. Adult women (≥ 18 years) were considered eligible for inclusion if they had completed a singleton pregnancy between 1 and 2017 and 1 January 2021 and undergone a 2-h 75-g oral glucose tolerance test (OGTT) during pregnancy. GDM was diagnosed based on the International Association of Diabetes and Pregnancy Study Groups criteria [26]. Women with incomplete information on childbirth, such as gestational age and birth weight, and those with an OGTT performed < 20 weeks or > 29 weeks of gestation were excluded from the study. Also excluded were women with pregestational diabetes or overt diabetes, since their earlier intensive interventions would have a significant impact on weight progression. We further excluded women with fewer than two weight measurements both in the second trimester and third trimester to ensure the accuracy of weight gain rate (WGR) calculations. According to the Chinese pre-pregnancy and pregnancy care guidelines, it is recommended that pregnant women attend 7–11 routine antenatal care visits throughout their pregnancy to assess potential risks regarding both the mother and the developing fetus. These visits are typically scheduled before the 13th week for the first time, and then at regular intervals of 14–19, 20–24, 25–28, 29–32, 33–36, and 37–41 weeks of gestational age [27]. Information on demographic characteristics, pre-existing health conditions, previous reproductive history, measured blood pressure, and self-reported pre-pregnancy weight and height were collected at the initial visit. During the following antenatal visits, maternal weight in lightweight clothing was routinely monitored, along with blood pressure, using the same set of calibrated electronic scales. Maternal baseline health records and longitudinal data including blood pressure, weight measurements, maternal complications, hospitalization before delivery, and childbirth were obtained from electronic medical records via a unique membership identifier. Ethical permission for this study was provided by the institutional review boards at Guangzhou Women and Children’s Medical Center. Given all maternal and neonatal data were extracted from the hospital EMR system by a unique identifier with no participant involved in the design, the written informed consent was waived.

### Outcome measures

Outcomes of interest were the average change in weight progression and adherence to gestation weight gain guidelines, which was assessed by total GWG and WGR in the second and third trimesters. To calculate GWG, we subtracted pre-pregnancy weight from weight at the end of pregnancy, independent of gestational age. We also calculated WGR before and after the OGTT accounting for changes in gestational age, based on serial weight measurements taken from the 13th week to the test, and from the test to delivery, respectively. To ensure the accuracy of WGR calculation, we took the initial weight measurement in the specified time window as the baseline weight for each individual, subtracted it from each subsequent weight, divided the results by the respective interval weeks as individual WGR, and averaged the WGRs to determine the final WGR in the predefined time window. The GWG and WGR were categorized into insufficient, adequate, and excessive according to Chinese weight guidelines [14].

### Other variables

The covariates included age at delivery, education, pre-pregnancy BMI, chronic hypertension, hospitalization before delivery, number of prenatal visits, gestational weeks for OGTT, gestational age, parity, and fetal sex. Chronic hypertension, defined as hypertension diagnosed or present before pregnancy or before 20 weeks of gestation, was identified based on systolic blood pressure ≥ 140 mm Hg or diastolic blood pressure ≥ 90 mm Hg, or both [28]. Pre-pregnancy BMI was calculated as weight in kilograms (kg) divided by height in meters squared (m^2^) and categorized into underweight (BMI < 18.5 kg/ m^2^), normal weight (BMI 18.5–23.9 kg/m^2^), overweight (BMI 24.0-27.9 kg/m^2^), and obese (BMI ≥ 28 kg /m^2^) based on Chinese BMI criteria [29]. In the stratified analysis by BMI, we included overweight and obese into one category as there were limited obese cases.

### Statistical analyses

We summarized the demographic and clinical variables for the whole cohort and compared subgroups with GDM and NGT using the student-t test for continuous variables or the chi-square test for categorical variables. We depicted weight progression throughout pregnancy and the corresponding density distribution of measured gestational age for both non-GDM and GDM populations. Furthermore, we ran a mixed effects model to assess the difference in weight progression between the two groups using a random intercept to account for repeated measures of the same individual. The fixed effects in the model included the presence of GDM, gestational weeks for weight measurements, and a multiplicative interaction term between the two variables. Covariates with a statistical difference between the groups were to be adjusted in the model, including maternal age, parity, pre-pregnancy BMI, chronic hypertension, and hospitalization before delivery. In stratification analyses by pre-pregnancy BMI, adjustments were further made for pre-pregnancy weight. Additionally, we compared the adherence to current weight recommendations between the two groups regarding total GWG, and WGR before and after the OGTT.

There were missing values for several variables including sex (29 [0.5%]), parity (29 [0.5%]), education (5368 [9.5%]), pre-pregnancy weight (4788 [8.5%]), and height (1620 [2.9%]), which resulted in a missing proportion of 10.1% (n = 5693) for pre-pregnant BMI. We used random imputation to address missing values for variables with low missing proportions, such as sex (male and female) and parity (primiparous and multiparous), based on their actual prevalence within the total cohort, which ensured that the imputed values were representative of the overall characteristics of the cohort. Missing values for education were considered a new category. For pre-pregnant weight and height, missing values were handled by multivariate imputation using chained equations (MICE) [30] based on the presence of GDM, and WGR before and after the OGTT. To test the robustness of our study, we conducted a sensitivity analysis by excluding those individuals with incomplete data on pre-pregnancy BMI, those with chronic hypertension, and those who were hospitalized before delivery. We further repeated the comparison for compliance with the IOM recommendations between GDM and NGT individuals. *P*-value of < 0.05 was considered statistically significant. All statistical analyses were performed using the statistical software program R, version 4.0.2.

## Results

**Fig. 1:**
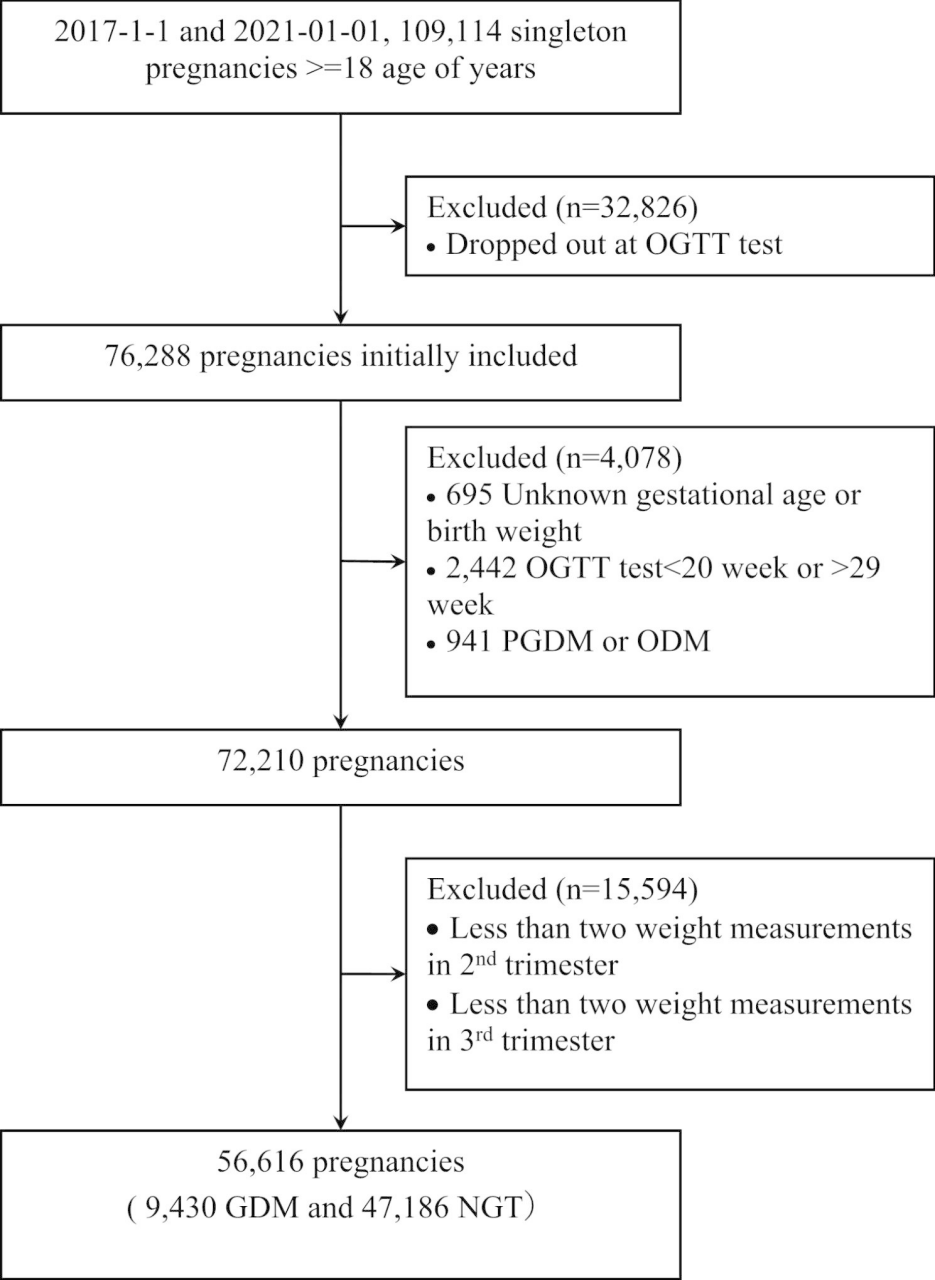
The flow chart of the study population. Abbreviations: GDM, gestational diabetes mellitus; NGT, normal glucose tolerance. Abbreviations: GDM, gestational diabetes mellitus; NGT, normal glucose tolerance

A total of 56,616 pregnancies (mean maternal age 30.9 [SD 4.3] years of age) were included in the main analysis, out of which 9,430 (16.7%) individuals were affected by GDM (Fig. 1). The characteristics of individuals with GDM and NGT are shown in Table 1. Individuals with GDM were older than NGT (32.5 [SD 4.4] years vs. 30. 6 [SD 4.2] years), more likely to be overweight/obese, and multiparous. Absolute weight change during gestation was 12.1 (SD 5.2) kg and 14.0 (SD 5.0) kg in individuals with GDM and NGT, respectively. However, individuals with GDM had a shorter gestation length than those with NGT (Table 1).

**Table 1:** Demographic and clinical characteristics of the study population

Variables	Total (N = 56,616)	GDM (n = 9,430)	NGT (n = 47,186)	*P* value	
Maternal age, years	30.9 (4.3)	32.5 (4.4)	30. 6 (4.2)	< 0.001	
18–25	4,896 (8.6)	348 (3.7)	4,548 (9.6)		
25–29	23,711 (41.9)	3,028 (32.1)	20,683 (43.8)		
30–34	19,581 (34.6)	3,675 (39.0)	15,906 (33.7)		
35–60	8,428 (14.9)	2,379 (25.2)	6,049 (12.8)		
Education level				0.41	
High school or below	7,526 (13.3)	1,279 (13.6)	6,247 (13.2)		
College or university	37,714 (66.6)	6,234 (66.1)	31,480 (66.7)		
Postgraduate	6,008 (10.6)	988 (10.5)	5,020 (10.6)		
Unknown	5,368 (9.5)	929 (9.9)	4,439 (9.4)		
Pre-pregnancy weight, kg	52.3 (6.7)	54.0 (7.1)	51.9 (6.5)	< 0.001	
Pre-pregnancy BMI, kg/m^2^	20.6 (2.6)	21.4 (2.8)	20.4 (2.5)	< 0.001	
Underweight	11,690 (20.6)	1,312 (13.9)	10,378 (22.0)		
Normal weight	39,420 (69.6)	6,495 (68.9)	32,925 (69.8)		
Overweight	4,967 (8.8)	1,440 (15.3)	3,527 (7.5)		
Obese	539 (1.0)	183 (1.9)	356 (0.8)		
Chronic hypertension, yes	1696 (3.0)	1309 (2.8)	387 (4.1)	< 0.001	
Hospitalization, yes	4531 (8.0)	3522 (7.5)	1009 (10.7)	< 0.001	
Number of antenatal visits	10.2 (2.5)	10.5 (2.5)	10.1 (2.5)	< 0.001	
Multiparous, yes	26,842 (47.4)	5,270 (55.9)	21,572 (45.7)	< 0.001	
Fetal Sex, female	26,807 (47.3)	4,453 (47.2)	22,354 (47.4)	0.80	
Weeks of OGTT	25.0 (1.3)	25.1 (1.3)	25.0 (1.3)	0.45	
Weeks of delivery	39.3 (1.2)	39.0 (1.2)	39.3 (1.2)	< 0.001	
Total GWG, kg	13.7 (5.1)	12.1 (5.2)	14.0 (5.0)	< 0.001	

Abbreviations: GDM, gestational diabetes mellitus; NGT, normal glucose tolerance; GWG, gestational weight gain

**Fig. 2:**
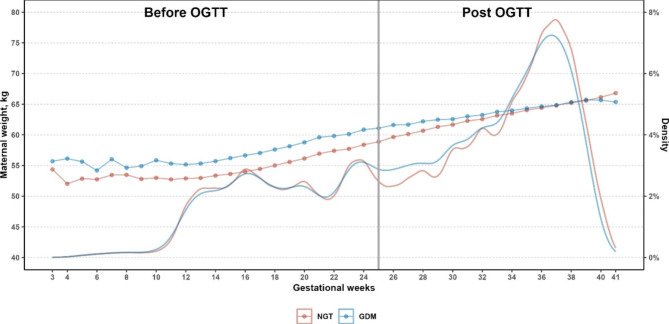
Weight progression and density distribution of measured gestational age among NGT and GDM populations throughout pregnancy. Abbreviations: GDM, gestational diabetes mellitus; NGT, normal glucose tolerance; OGTT, oral glucose tolerance test

Figure 2 presents a visualization of weight progression throughout pregnancy and the corresponding density distribution of measured gestational age. The distribution of measured gestational age was similar between GDM and NGT groups, and weight progression seemed aligned before the OGTT, however, individuals with GDM exhibited a lower rate of weight gain thereafter. Table 2 provides estimates of average change in weight progression among GDM and NGT individuals during pregnancy, before and after the OGTT, respectively. Throughout gestation, GDM individuals gained weight at a slower rate of 0.39 kg per week than NGT individuals (0.51 kg/week) (Table 2). Before the OGTT, WGRs were barely different between GDM (0.44 kg/week) and NGT individuals (0.45 kg/week). However, individuals experienced a substantial decrease in the rate of weight gain following the diagnosis of GDM compared with NGT individuals (0.34 vs. 0.50 kg/week), which was consistently shown across different BMI categories, including underweight class (0.39 vs. 0.51 kg/week), normal weight class (0.35 vs. 0.50 kg/week) and overweight/obese class (0.29 vs. 0.45 kg/week). These results were also observed in the sensitivity analysis (Table S1).

**Table 2:** The coefficients for weight progression between women with gestational diabetes mellitus (GDM) and women with normal glucose tolerance (NGT) based on the mixed effects model

Time windows	GDM group			NGT group		Difference (95%CI)	*P* value	
	No. of weight measurements	*β* (95% CI), kg/week		No. of weight measurements	*β* (95% CI), kg/week			
**Overall** ^a^								
Total gestation	98, 850	0.39 (0.39, 0.39)		480, 007	0.51 (0.51, 0.51)	0.12 (0.12, 0.12)	< 0.001	
Before OGTT	32, 330	0.44 (0.43, 0.44)		159, 966	0.45 (0.45, 0.46)	0.02 (0.01, 0.02)	< 0.001	
After OGTT	66, 520	0.34 (0.34, 0.34)		320, 041	0.50 (0.50, 0.50)	0.16 (0.16, 0.16)	< 0.001	
**Underweight** ^b^								
Total gestation	13, 834	0.43 (0.42, 0.43)		105, 416	0.52 (0.52, 0.52)	0.09 (0.09, 0.09)	< 0.001	
Before OGTT	4, 618	0.46 (0.45, 0.47)		35, 199	0.47 (0.46, 0.47)	0.01 (0, 0.02)	< 0.001	
After OGTT	9, 216	0.39 (0.39, 0.40)		70, 217	0.51 (0.51, 0.51)	0.12 (0.11, 0.12)	< 0.001	
**Normal weight** ^b^								
Total gestation	68, 169	0.40 (0.39, 0.40)		336, 581	0.51 (0.51, 0.51)	0.12 (0.12, 0.12)	< 0.001	
Before OGTT	22, 241	0.45 (0.44, 0.45)		111, 749	0.46 (0.46, 0.46)	0.01 (0.01, 0.02)	< 0.001	
After OGTT	45, 928	0.35 (0.34, 0.35)		22, 4832	0.50 (0.50, 0.50)	0.16 (0.15, 0.16)	< 0.001	
**Overweight/obese** ^b^								
Total gestation	16, 847	0.33 (0.32, 0.33)		38, 010	0.45 (0.44, 0.45)	0.12 (0.11, 0.12)	< 0.001	
Before OGTT	5, 471	0.39 (0.38, 0.40)		13, 018	0.39 (0.38, 0.39)	-0.01 (-0.02, 0.01)	0.16	
After OGTT	11, 376	0.29 (0.28, 0.29)		24, 992	0.45 (0.44, 0.45)	0.16 (0.15, 0.17)	< 0.001	

Abbreviations: GDM, gestational diabetes mellitus; NGT, normal glucose tolerance. ^a^*β*, indicating the average change in weight progression, was estimated using a mixed effects model with adjustment for maternal age, parity, pre-pregnancy BMI, chronic hypertension, and hospitalization before delivery. ^b^*β* was estimated using a mixed effects model with adjustment for maternal age, parity, and pre-pregnancy weight.

**Fig. 3:**
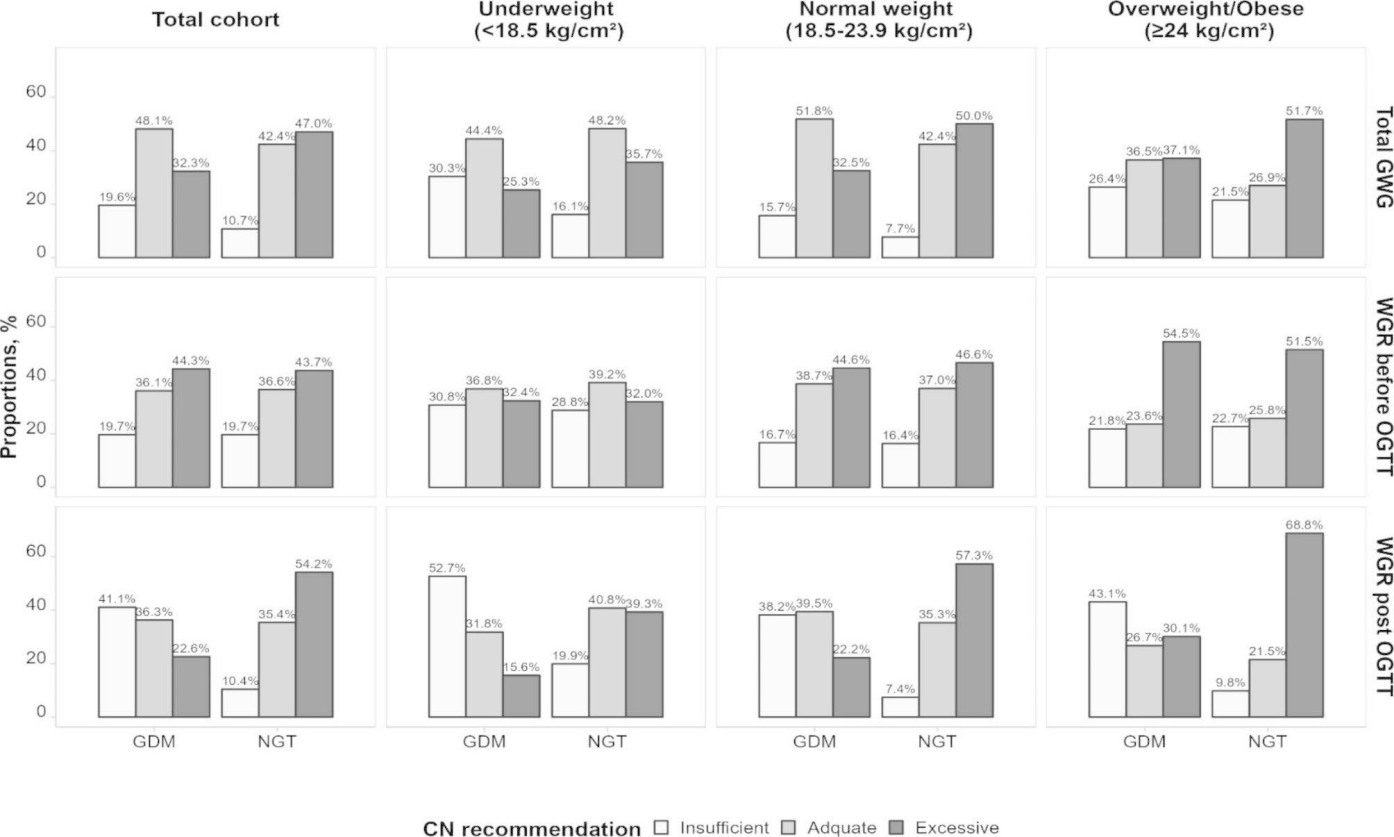
Adherence to Chinese gestational weight gain recommendations among women with GDM and NGT, stratified by pre-pregnancy BMI. Abbreviations: GDM, gestational diabetes mellitus; NGT, normal glucose tolerance; GWG, gestational weight gain; WGR, weight gain rate

By absolute weight change, the percentages of insufficient GWG were 19.6% and 10.7% between GDM and NGT individuals, while those of excessive GWG were 32.3% and 47.0%, respectively (Fig. 3). This was more evident after the OGTT when using WGR as an indicator, with a four-fold increased percentage of insufficient weight gain and a two-fold decreased percentage of excessive weight gain in GDM individuals as compared to NGT individuals (insufficient, 41.1% vs. 10.4%; excessive, 22.6% vs. 54.2%), including in normal weight class (insufficient, 38.2% vs. 7.4%; excessive, 22.2% vs. 57.3%) and overweight/obese class (insufficient, 43.1% vs. 9.8%; excessive, 30.1% vs. 68.8%) (Fig. 3). In the second and third trimesters before OGTT, however, there was no difference in the distribution of insufficient and excessive weight gain between GDM and NGT individuals. Per IOM recommendations, while similar results were observed, the proportion of insufficient GWG was remarkably higher both in GDM and NGT groups (Figure S1).

## Discussion

In this longitudinal hospital-based study of pregnant women, GDM individuals experienced different weight progression patterns from NGT individuals, manifesting as gaining weight at a similar rate before the OGTT but at a significantly slower rate after the test. Consequently, per current gestational weight gain recommendations, GDM individuals presented a considerably higher incidence of insufficient weight gain and a reduced proportion of excessive weight gain as compared to NGT individuals. These findings indicate the need for distinct weight gain recommendations for GDM and NGT populations that take into account their differences in weight progression patterns.

Several previous studies have assessed the GWG between women with GDM and women with NGT. In a birth cohort of 3260 Finnish pregnant women, GDM individuals had a GWG of 9.4 kg through gestation as opposed to 12.6 kg in NGT individuals [31]. Also, in a prospective study of 212 Australian women (115 GDM and 97 NGT), GDM individuals gained more weight before OGTT (8.4 vs. 7.5 kg) but gained less thereafter, in comparison to NGT individuals (1.18 vs. 4.0 kg) [32]. Our data, along with those from previous studies, consistently indicated a substantial difference in weight gain profiles between GDM and NGT individuals. Additionally, we were able to compare the average change in weight progression based on serial weight measurements during pregnancy. We found a similar average rate of weight gain between GDM and NGT individuals before the OGTT, however, there was a substantial decrease in the average rate of weight gain after the test among GDM individuals in comparison to NGT individuals. Individuals affected by GDM are typically offered comprehensive health advice on achieving glycemic goals, including nutrition, physical activity, lifestyle and behavioral modification, and glucose monitoring [18]. Such structured interventions would result in a significant reduction in nutrients and energy, which may be the main explanation for the decreased weight gain in GDM individuals [31, 33, 34].

In this study, less than half of the pregnant women met the current weight gain targets, which was consistent with previous studies, reporting a compliance rate of 23–51% worldwide [35], including 25–35% in those with GDM [20, 36, 37]. However, as a result of less weight gain, GDM individuals were more prone to be labeled as having insufficient weight gain instead of being identified as having excessive weight gain in comparison to NGT individuals. Likewise, Zheng et al. found the percentage of women with GDM who gained weight below the recommendations doubled before and after the OGTT (21% vs. 41%) [8]. Komem et al. also reported a considerable increase in the percentage of women with insufficient GWG before and after GDM diagnosis (42.9% vs. 70%) [20]. From the perspective of weight management, women with insufficient weight gain require appropriate increases in nutrition and energy to achieve the established weight targets, however, this is in turn possible to hinder their later glycemic control interventions. This discrepancy between weight management and glycemic control points to the need to distinguish between NGT individuals and GDM individuals, who may require lower weight targets than they currently have. Gestational weight gain is significantly correlated with a decrease in insulin sensitivity and excessive GWG following GDM diagnosis may exaggerate insulin sensitivity and the progress of GDM to DM [38–40]. In this sense, failing to identify women with potential excessive weight gain may cause additional health risks. Recently, several studies found that GDM individuals with GWG below the weight recommendations were at a significantly decreased risk of fetal macrosomia, and those who lost weight during GDM management did not increase the risk of the small-for-gestational-age infant compared to those above IOM targets [8, 41–45], adding further evidence supporting the benefit of lower weight targets for women with GDM.

The main strength of this study was the thorough use of serial weight measurements. Given that GDM individuals were more likely to have a shorter length of gestation in comparison to NGT individuals, the less weight gain observed in individuals with GDM may be partly influenced by the residual bias of gestation length. Based on the serial weight measurements, we were able to evaluate the average change in weight progression within different gestation windows, which was independent of gestation length and may be more effective than the total GWG in weight management as a result. In addition, each woman undergoes scheduled obstetric care, and maternal weight was measured using a unified scale and captured timely by the EMR system, therefore, measurement bias and recall bias was largely avoided. Third, the large study population was also an important strength of this study, which enables us to clarify the group differences across different BMI classes. The proportion of overweight/obese BMI among GDM individuals was higher than that among NGT individuals, and pregnant women with overweight/obesity generally gained less weight during pregnancy, therefore, analysis stratified by BMI was possible to avoid the confounding bias by BMI. Our study also had several limitations. First, we do not have data on dietary patterns, nutrient intake, and physical activity, which might help to further elucidate the mediating or moderating effects of these factors. Second, there was about 10% of missing data with pre-pregnancy weight. While we used a multiple imputation method to assign the missing values, some degree of misclassification was possible for pre-pregnancy BMI. However, we repeated the main analysis excluding patients with missing data, and the results did not alter. Third, the population is dominated by the Han ethnicity living in Guangzhou, thus it may potentially limit the generalizability of the findings to other races/ethnicities.

## Conclusions

Weight progression varied significantly between GDM and NGT individuals, resulting in a substantial difference in identifying insufficient and excessive weight gain between the two groups under the current gestational weight gain guidelines. These findings indicate the need for developing distinct weight gain recommendations for GDM and NGT populations in future clinical practice, and lower weight gain targets may be more appropriate for GDM individuals given the requirement for glucose management.

## Electronic supplementary material

Below is the link to the electronic supplementary material.

Supplementary Material 1

## Data Availability

The datasets generated and analyzed during the current study are not publicly available due to ethical concerns but are available from the corresponding author on reasonable request.
