# Supplementary material for: Weight progression and adherence to weight gain target in women with vs. without gestational diabetes: a retrospective cohort study
Source: BMC Pregnancy Childbirth. 2023 Jul 13;23:513. doi: 10.1186/s12884-023-05832-x (PMC10347803; doi:10.1186/s12884-023-05832-x)
Supplement: Supplementary file 1 — Supplementary Material 1 [file 12884_2023_5832_MOESM1_ESM.docx]

**Table S1. Sensitivity analysis for difference in weight progression between individuals with gestational diabetes mellitus (GDM) and with normal glucose tolerance (NGT)**

| **Time windows** | **GDM group** | |  | **NGT group** | | **Difference (95%CI)** | ***P* value** |
| --- | --- | --- | --- | --- | --- | --- | --- |
|  | **No. of weight measurements** | ***β* (95% CI), kg/week** |  | **No. of weight measurements** | ***β* (95% CI), kg/week** |  |  |
| **Overall** ^a^ |  |  |  |  |  |  |  |
| Total gestation | 75,961 | 0.39 (0.39, 0.39) |  | 390,417 | 0.51 (0.51, 0.51) | 0.12 (0.12, 0.12) | <0.001 |
| Before OGTT | 24,858 | 0.44 (0.44, 0.44) |  | 130,059 | 0.46 (0.45, 0.46) | 0.02 (0.01, 0.02) | <0.001 |
| After OGTT | 51,103 | 0.35 (0.34, 0.35) |  | 260,358 | 0.50 (0.50, 0.50) | 0.15 (0.15, 0.16) | <0.001 |
| **Underweight** ^b^ |  |  |  |  |  |  |  |
| Total gestation | 10,367 | 0.43 (0.42, 0.43) |  | 86,292 | 0.51 (0.51, 0.52) | 0.09 (0.08, 0.09) | <0.001 |
| Before OGTT | 3,488 | 0.45 (0.44, 0.47) |  | 28,864 | 0.47 (0.46, 0.47) | 0.01 (0, 0.02) | 0.04 |
| After OGTT | 6,879 | 0.39 (0.39, 0.40) |  | 57,428 | 0.51 (0.51, 0.51) | 0.12 (0.11, 0.12) | <0.001 |
| **Normal weight** ^b^ |  |  |  |  |  |  |  |
| Total gestation | 52,870 | 0.40 (0.40, 0.40) |  | 274,628 | 0.51 (0.51, 0.51) | 0.11 (0.11, 0.12) | <0.001 |
| Before OGTT | 17,298 | 0.45 (0.45, 0.46) |  | 91,116 | 0.46 (0.46, 0.46) | 0.01 (0.01, 0.02) | <0.001 |
| After OGTT | 35,572 | 0.35 (0.35, 0.36) |  | 183,512 | 0.50 (0.50, 0.50) | 0.15 (0.15, 0.16) | <0.001 |
| **Overweight/obese** ^b^ |  |  |  |  |  |  |  |
| Total gestation | 12,724 | 0.33 (0.32, 0.33) |  | 29,497 | 0.45 (0.44, 0.45) | 0.12 (0.11, 0.12) | <0.001 |
| Before OGTT | 4,072 | 0.39 (0.37, 0.4) |  | 10,079 | 0.39 (0.38, 0.40) | 0 (-0.01, 0.02) | 0.53 |
| After OGTT | 8,652 | 0.29 (0.28, 0.30) |  | 19,418 | 0.44 (0.44, 0.45) | 0.15 (0.14, 0.16) | <0.001 |
| Abbreviations: GDM, gestational diabetes mellitus; NGT, normal glucose tolerance. ^a^ *β,* indicating the rate of weight progression, was estimated using a mixed effects model with adjustment for maternal age, parity, pre-pregnancy BMI. ^b^ *β* was estimated using a mixed effects model with adjustment for maternal age, parity, and pre-pregnancy weight. | | | | | | | |


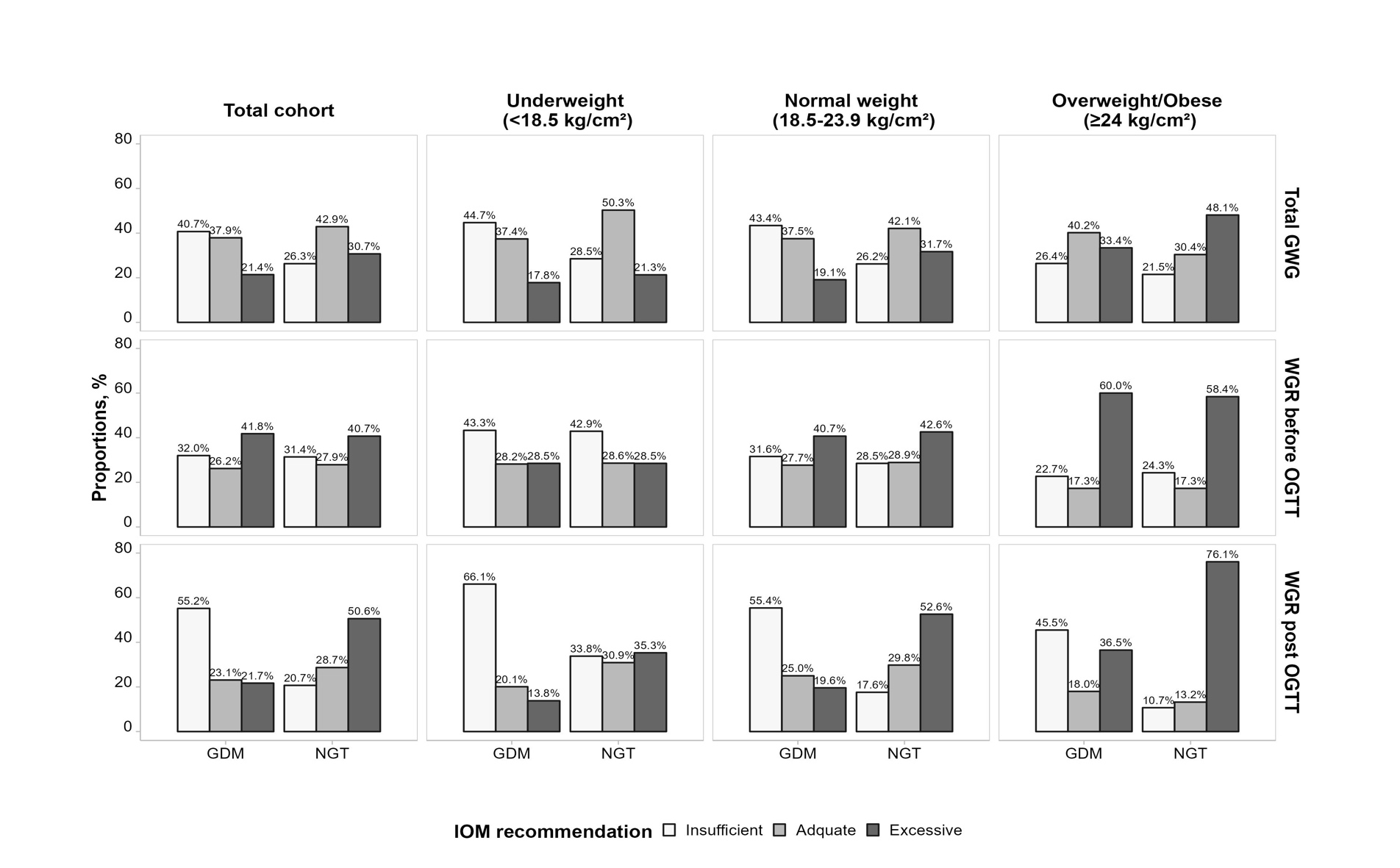


Figure S1. Adherence to IOM recommendations among women with GDM and NGT, stratified by pre-pregnancy BMI. Abbreviations: GDM, gestational diabetes mellitus; NGT, normal glucose tolerance; GWG, gestational weight gain; WGR, weight gain rate.
